# Circulating lipocalin‐2 as a novel biomarker for early neurological deterioration and unfavorable prognosis after acute ischemic stroke

**DOI:** 10.1002/brb3.2979

**Published:** 2023-03-27

**Authors:** Yi Xie, Xingfeng Zhuo, Kai Xing, Zhenqian Huang, Hongquan Guo, Pengyu Gong, Xiaohao Zhang, Yun Li

**Affiliations:** ^1^ Department of Neurology Jinling Hospital, Medical School of Nanjing University Nanjing Jiangsu China; ^2^ Outpatient Department Jinling Hospital, Medical School of Nanjing University Nanjing Jiangsu China; ^3^ Department of Neurology Affiliated Hospital of Nantong University Nantong Jiangsu China; ^4^ Department of Neurology Nanjing First Hospital, Nanjing Medical University Nanjing Jiangsu China

**Keywords:** biomarker, clinical worsening, functional outcome, ischemic stroke, LCN2

## Abstract

**Introduction:**

Lipocalin‐2 (LCN2) is an acute‐phase protein that could mediate neuroinflammation after brain injury. We aimed to evaluate if LCN2 level was associated with early neurological deterioration (END) in acute ischemic stroke patients, thus hindering clinical recovery.

**Methods:**

We conducted a prospective study of acute ischemic stroke patients between June 2021 and February 2022. Serum LCN2 concentration was measured after admission using an enzyme‐linked immunosorbent assay. Outcomes included END and 90‐day poor functional outcome (modified Rankin Scale 3‐6). The National Institutes of Health Stroke Scale increment ≥4 points within 72 h after admission was defined as END.

**Results:**

A total of 253 acute ischemic stroke patients (mean age, 65.2 ± 13.4 years; 64.0% male) were recruited. In the multivariate adjustment, increased serum LCN2 levels (per 1‐SD increase of LCN2) were associated with a higher risk of END (odds ratio [OR], 1.64; 95% confidence interval [CI], 1.20–2.25; *p* = .002) and 90‐day poor outcome (OR, 1.73; 95% CI, 1.22–2.45; *p* = .002). Restricted cubic splines found a linear relationship between LCN2 level and 90‐day unfavorable outcome (END, *p* = .001 for linearity; 90‐day poor outcome, *p* = .013 for linearity). Subgroup analysis further confirmed the significant association of LCN2 with clinical outcomes.

**Conclusions:**

This study demonstrated that higher circulating LCN2 level was associated with an increased risk of early clinical worsening and 90‐day unfavorable outcomes in ischemic stroke patients.

## Graphical abstract

1. END was a common complication after ischemic stroke; 2. Higher LCN2 levels were found in patients with END and 90 days unfavorable outcome? 3. LCN2 may involve in the pathophysiology of clinical worsening after stroke.

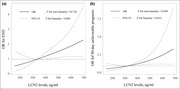

## 1 INTRODUCTION

Stroke is the second leading cause of adult disability and mortality worldwide (GBD 2019 Stroke Collaborators, 2021). Despite advances in disease prevention and acute management in China, the stroke burden is expected to increase in the past 7 years (2013–2019) (Tu et al., 2022). Some patients experience neurological worsening during the acute phase, namely, early neurological deterioration (END), with a prevalence ranging from 5% to 40% (Siegler & Martin‐Schild, 2011; Thanvi et al., 2008; Zhang et al., 2016). Several studies demonstrated early or subacute neurological deterioration might have a deleterious impact on functional recovery (Liu et al., 2020; Mori et al., 2012). Therefore, rapid prediction of clinical outcomes at the acute phase of ischemic stroke is of vital importance for prognosis improvement.

Lipocalin‐2 (LCN2) is an acute‐phase protein of the lipocalin family and is highly expressed in response to brain injury and inflammatory stimuli (Dekens et al., 2018; Naudé et al., 2012; Xiao et al., 2017; Zhao et al., 2019). The LCN2 protein was primarily expressed in astrocytes and endothelial cells after focal cortical ischemia in mice (Wan et al., 2022). In a previous animal study of the transient middle cerebral artery occlusion model, LCN2 inhibition could alleviate ischemic brain damage, including amelioration of neuroinflammation and blood–brain barrier disruption (Jin et al., 2014). Moreover, in vitro studies demonstrated that LCN2 deficiency in astrocytes could alleviate direct neurotoxic effects on neurons under oxygen and glucose‐deprived conditions (Suk, 2016). A previous clinical study found that LCN2/MMP‐9 complex concentrations could be used to identify unstable atherosclerotic plaques and major adverse cardiovascular events (Cheng et al., 2014; Hemdahl et al., 2006). However, few data are available to date regarding the prognostic value of serum LCN2 in ischemic stroke patients. Herein, we performed a prospective study and aimed to evaluate whether circulating LCN2 levels were correlated with clinical outcomes in patients with ischemic stroke.

## 2 METHODS

### 2.1 Study population

Patients diagnosed with first‐ever ischemic stroke and hospitalized within 72 h after symptoms onset were prospectively enrolled in Jinling Hospital during June 2021 and February 2022. The exclusion criteria of this study were as follows: (1) age <18 years; (2) early discharged within 3 days after admission; (3) pre‐stroke modified Rankin Scale (mRS) score >2; (4) had severe pulmonary disease, renal and liver failure, and active malignant. This study protocol was reviewed and approved by the ethics committee of Jinling Hospital (2021DZGZR‐YBB‐115). All subjects or their legally authorized representatives signed informed consent before entering the study.

### 2.2 Baseline data collection

Baseline data were collected after admission by trained neurologic clinicians. These data included age, sex, vascular risk factors, baseline stroke severity, stroke subtype, infarct volume, and white matter lesions (WMLs). Stroke severity was evaluated by the National Institutes of Health Stroke Scale (NIHSS) (Brott et al., 1989). Pretreatment infarction core was assessed by the Alberta stroke program early computerized tomography (ASPECT) score (Barber et al., 2000). Stroke etiology was defined according to the criteria of Trial of Org 10172 in Acute Stroke Treatment (TOAST) (Adams et al., 1993). We measured the WMLs in the hemisphere contralateral to acute stroke using the Fazekas scale (Fazekas et al., 1987). The total WMLs score was calculated by summing up the scores for subcortical WMLs and periventricular WMLs, ranging from 0 to 6. According to previous studies, severe WMLs were defined as a total WMLs score ≥3 (Kim et al., 2014; Yakushiji et al., 2014).

### 2.3 LCN2 level assessment

The blood samples were collected within 24 h after admission and processed under standard laboratory procedure. Serum samples were stored at −80°C for further analysis. The serum LCN2 levels were measured using the ELISA Kit (Cat. EK0853; Boster Biological Technology, Wuhan, China). The intra‐ and inter‐assay coefficients of variation were <7.5% and <7.5%, respectively. The minimum detectable concentration was 10.0 pg/mL. All samples were measured by a laboratory technician who was blinded to any clinical information of the study participants.

### 2.4 Clinical outcome assessment

The clinical outcomes included END and functional outcomes at 90 days. The neurological deficit was evaluated using the NIHSS at baseline and continued 1–3 times a day for 72 h by a certified neurologist, who was blind to clinical information. In our study, END was defined as a total NIHSS score ≥4 points deterioration within 72 h after admission (Alawneh et al., 2009; Sun et al., 2014). The 3‐month follow‐up was conducted via telephone or outpatient clinic using the mRS. The functional outcome was dichotomized as favorable (mRS 0–2) and unfavorable (mRS 3–6).

### 2.5 Statistical analysis

Data were presented as the mean ± standard deviations (SD) or median with interquartile range (IQR) for continuous variables and as percentages for categorical variables. Univariate analyses were utilized using the Fisher exact test or *χ* ^2^ test for qualitative variables, and the *t*‐test or Mann–Whitney *U* test for quantitative variables, where appropriate. Logistic regression analyses were used to assess the associations of LCN2 levels with END and functional outcomes. Then, age, sex, and variables with a *p* value <.1 in univariate analysis were adjusted in the multivariate logistic regression analysis. Subgroup analysis was conducted to test the robustness of our findings. Odds ratios (ORs) with 95% confidence intervals (CIs) were calculated. We further evaluated the pattern and magnitude of the association between LCN2 level and clinical outcomes using the restricted cubic splines with 3 kn (at 5th, 50th, and 95th percentiles) adjusted for covariates (Durrleman & Simon, 1989). All analyses were conducted using statistical software SPSS version 24.0 (IBM, New York, NY, USA) and R statistical software (R, version 4.1; R Project). A 2‐sided *p* value <.05 was considered to be statistically significant.

## 3 RESULTS

In this study, 273 ischemic patients hospitalized within 72 h after symptoms onset were screened for analysis. We excluded four patients early discharged within 72 h, three patients had a pre‐stroke mRS score >2, seven patients diagnosed with active malignant, and six patients lost in the follow‐up. Finally, a total of 253 patients were enrolled. Finally, a total of 253 patients were analyzed, among whom 162 (64.0%) patients were male. The mean age was 65.2 ± 13.4 years. Hypertension was present in 176 (69.6%), diabetes mellitus in 87 (34.4%), hyperlipidemia in 34 (13.4%), and severe WMLs in 129 (51.0%) patients. Fifty‐one (20.2%) patients received reperfusion therapy after admission. The median NIHSS score at admission was 4.0 points. The median serum LCN2 concentration was 330.0 ng/mL (IQR, 258.6–500.4 ng/mL). The difference in clinical data stratified by the quartile of LCN2 was demonstrated in Table 1. Increased LCN2 levels showed a significant correlation with severe WMLs (*p* = .021) and hypersensitive C‐reactive protein (*p* = .024).

**TABLE 1:** Comparison of baseline data stratified by the lipocalin‐2 (LCN2) quartile

Variable	1st quartile (*n* = 63)	2nd quartile (*n* = 62)	3rd quartile (*n* = 64)	4th quartile (*n* = 64)	*p* Value
Demographic characteristics					
Age (years)	63.5 ± 15.5	64.1 ± 13.9	65.4 ± 11.4	68.0 ± 11.5	.491
Male, *n* (%)	40 (63.5)	40 (64.5)	44 (68.8)	38 (59.4)	.745
Body mass index (kg/m^2^)	24.9 ± 3.2	24.6 ± 3.5	25.2 ± 3.5	25.0 ± 3.1	.837
Vascular risk factors, *n* (%)					
Hypertension	45 (71.4)	43 (69.4)	42 (65.6)	46 (71.9)	.865
Diabetes mellitus	20 (31.7)	23 (37.1)	19 (29.7)	25 (39.1)	.650
Hyperlipidemia	8 (12.7)	8 (12.9)	9 (14.3)	9 (14.1)	.993
Coronary heart disease	9 (14.3)	6 (9.7)	9 (14.3)	3 (4.7)	.243
Smoking	21 (33.3)	20 (32.3)	28 (43.8)	22 (34.4)	.510
Clinical data					
Previous statin therapy, *n* (%)	9 (14.3)	17 (27.4)	12 (18.8)	10 (5.6)	.236
Previous antiplatelet therapy, *n* (%)	10 (15.9)	16 (25.8)	14 (21.9)	14 (21.9)	.598
Systolic blood pressure (mmHg)	149.3 ± 26.8	144.7 ± 23.0	143.1 ± 19.6	147.6 ± 26.0	.344
Diastolic blood pressure (mmHg)	81.9 ± 15.0	82.4 ± 14.4	83.5 ± 12.0	84.8 ± 15.0	.556
Baseline NIHSS, score	3.0 (2.0, 10.0)	3.0 (2.0, 8.0)	4.0 (2.0, 8.0)	4.0 (2.0, 9.0)	.754
Severe white matter lesions	23 (36.5)	32 (51.6)	33 (51.6)	41 (64.1)	.021
Site of infarction, *n* (%)					.346
Anterior circulation	50 (79.4)	46 (24.2)	56 (87.5)	54 (84.4)	.236
Posterior circulation	14 (22.2)	16 (25.8)	9 (14.1)	10 (15.6)	.295
Cause of stroke, *n* (%)					.968
Atherosclerotic	21 (33.3)	23 (37.1)	26 (40.6)	26 (40.6)	
Cardioembolic	10 (15.9)	12 (19.4)	11 (17.2)	8 (12.5)	
Small vessel occlusion	22 (34.9)	18 (29.0)	17 (26.6)	22 (34.4)	
Others	10 (15.9)	9 (14.5)	10 (15.6)	8 (12.5)	
Laboratory data					
Blood urea nitrogen/creatinine	13.9 ± 4.7	14.0 ± 4.5	12.8 ± 4.0	13.7 ± 4.4	.432
Homocysteine (mmol/L)	11.5 (10.0, 14.9)	12.9 (9.7, 15.4)	12.7 (9.8, 16.4)	12.4 (10.2, 16.9)	.980
Baseline blood glucose (mmol/L)	5.7 (4.9, 7.7)	6.0 (5.0, 9.3)	6.1 (5.0, 7.9)	6.3 (5.0, 8.2)	.576
Hs‐CRP (mg/L)	3.2 (1.2, 7.5)	4.3 (1.5, 10.3)	5.3 (1.9, 9.3)	8.8 (1.6, 14.5)	.024

Abbreviations: Hs‐CRP, hypersensitive C‐reactive protein; NIHSS, National Institutes of Health Stroke Scale.

Table 2 demonstrates the baseline data of the study population stratified by the clinical outcomes. During the hospitalization, 41 (16.2%) subjects experienced END, and 18 (7.2%) patients died. As compared to patients without END, patients with END were more likely to develop diabetes (*p* = .034) and had higher baseline NIHSS score (*p* = .009) and LCN2 levels (*p* = .021). During the 90‐day follow‐up, 116 (45.8%) patients experience unfavorable outcomes (mRS score of 3–6). Age (*p* = .044), baseline NIHSS score (*p* = .001), severe WMLs (*p* = .025), and LCN2 levels (*p* = .010) differed significantly between patients with and without poor outcome.

**TABLE 2:** Comparison of baseline data stratified by clinical outcomes

Variable	All patients (*n* = 253)	With END (*n* = 41)	Without END (*n* = 212)	*p* Value	Unfavorable outcome (*n* = 116)	Favorable outcome (*n* = 137)	*p* Value
Demographic characteristics							
Age (years)	65.2 ± 13.4	67.7 ± 11.8	64.7 ± 13.7	.182	67.0 ± 12.6	63.6 ± 14.0	.044
Male, *n* (%)	162 (64.0)	29 (70.7)	133 (62.7)	.954	76 (65.6)	86 (62.8)	.650
Body mass index (kg/m^2^)	24.9 ± 3.3	24.9 ± 4.1	24.9 ± 3.2	.951	25.0 ± 3.7	24.9 ± 3.0	.731
Vascular risk factors, *n* (%)							
Hypertension	176 (69.6)	29 (70.7)	147 (69.3)	.859	79 (68.1)	97 (70.8)	.642
Diabetes mellitus	87 (34.4)	20 (48.8)	67 (31.6)	.034	40 (34.5)	47 (34.3)	.977
Hyperlipidemia	34 (13.4)	5 (12.2)	29 (13.7)	.799	13 (11.2)	21 (15.3)	.338
Coronary heart disease	27 (10.7)	3 (7.3)	24 (11.4)	.442	13 (11.2)	14 (10.2)	.781
Smoking	91 (36.0)	16 (39.0)	75 (35.4)	.656	41 (35.3)	50 (36.5)	.849
Clinical data							
Previous statin therapy, *n* (%)	48 (19.0)	5 (12.2)	43 (20.3)	.227	18 (15.5)	30 (21.9)	.197
Previous antiplatelet therapy, *n* (%)	54 (12.3)	9 (22.0)	45 (21.2)	.917	23 (19.8)	31 (22.6)	.588
Systolic blood pressure (mmHg)	146.2 ± 24.0	148.6 ± 20.6	145.6 ± 24.6	.476	145.5 ± 23.1	146.7 ± 24.8	.666
Diastolic blood pressure (mmHg)	83.2 ± 14.1	85.6 ± 13.2	82.7 ± 14.2	.224	82.4 ± 13.6	83.8 ± 14.6	.458
Baseline NIHSS, score	4.0 (2.0, 9.0)	7.0 (3.5, 10.0)	3.0 (2.0, 8.0)	.009	8.0 (4.0, 12.0)	2.0 (1.0, 4.0)	.001
Severe white matter lesions	129 (51.0)	26 (63.4)	103 (48.6)	.082	68 (58.6)	61 (44.5)	.025
Site of infarction, *n* (%)							
Anterior circulation	206 (81.4)	32 (78.0)	174 (82.1)	.544	94 (81.0)	112 (81.8)	.848
Posterior circulation	49 (19.4)	9 (22.0)	40 (18.9)	.647	24 (20.7)	25 (18.2)	.624
Cause of stroke, *n* (%)				.481			.130
Atherosclerotic	96 (37.9)	14 (34.1)	82 (38.7)		47 (40.5)	49 (35.8)	
Cardioembolic	41 (16.2)	10 (24.4)	31 (14.6)		24 (20.7)	17 (12.4)	
Small vessel occlusion	79 (31.2)	12 (29.3)	67 (31.6)		32 (27.6)	47 (34.3)	
Others	37 (14.6)	5 (12.2)	32 (15.1)		13 (11.2)	24 (17.5)	
Laboratory data							
Blood urea nitrogen/creatinine	13.6 ± 4.4	14.6 ± 5.6	13.4 ± 4.1	.124	13.9 ± 4.6	13.3 ± 4.2	.261
Homocysteine (mmol/L)	12.5 (10.0, 15.4)	11.6 (9.7, 14.1)	12.8 (10.0, 15.5)	.229	12.5 (10.0, 15.5)	12.8 (10.1, 15.4)	.661
Baseline blood glucose (mmol/L)	6.0 (5.0, 8.1)	7.0 (4.9, 8.7)	6.0 (5.0, 7.8)	.501	6.6 (5.0, 8.6)	5.7 (4.9, 7.3)	.102
Hs‐CRP (mg/L)	5.0 (1.5, 10.4)	6.2 (1.0, 13.4)	5.0 (1.5, 10.3)	.494	6.3 (1.4, 14.2)	4.6 (1.5, 8.6)	.177
LCN2 (ng/mL)	330.0 (258.6, 500.4)	414.4 (296.0, 571.6)	316.2 (248.3, 494.3)	.021	382.6 (281.5, 545.3)	314.7 (237.0, 466.3)	.010
LCN2 quartiles, *n* (%)				.089			.154
First	63 (24.9)	4 (9.8)	59 (27.8)		24 (20.7)	39 (28.5)	
Second	62 (24.5)	12 (29.3)	50 (23.6)		25 (21.6)	37 (27.0)	
Third	64 (25.3)	11 (26.8)	53 (25.0)		31 (26.7)	33 (24.1)	
Fourth	64 (25.3)	14 (34.1)	50 (23.6)		36 (31.0)	28 (20.4)	

Abbreviations: END, early neurological deterioration; Hs‐CRP, hypersensitive C‐reactive protein; LCN2, lipocalin‐2; NIHSS, National Institutes of Health Stroke Scale.

**FIGURE 1:**
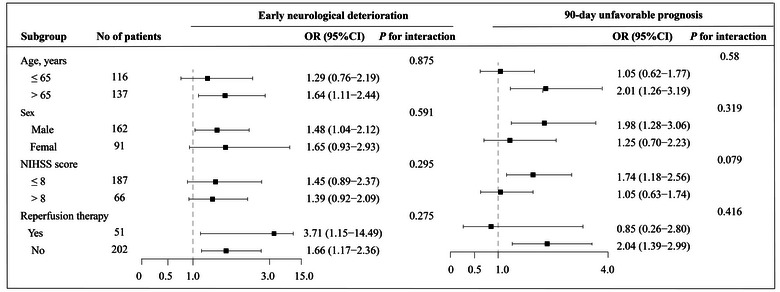
Subgroup analyses of the association between serum lipocalin‐2 (LCN2) and outcomes after ischemic stroke. Odds ratios (ORs) were calculated for each standard deviation (SD) increase in serum LCN2 levels after adjustment for age, sex, and variables with a *p* value <.1 in the univariate analysis, except for the stratified variable.

After adjusting for age, sex, and variables with a *p* value <.1 in univariate analysis, the multivariate regression analysis model showed that higher LCN2 levels were associated with an increased risk of END (per 1‐SD increase, OR, 1.64; 95% CI, 1.20–2.25; *p* = .002) and 90‐day poor outcome (per 1‐SD increase, OR, 1.73; 95% CI, 1.22–2.45; *p* = .000) (Table 3). In addition, the association of serum LCN2 with risk of END and the 90‐day unfavorable prognosis was similar across subgroups stratified according to age, sex, admission NIHSS score, and receiving reperfusion therapy (*p* > .05 for interaction for all; Figure 1).

**TABLE 3:** Multivariate analysis of the association between lipocalin‐2 (LCN2) levels and clinical outcome

Variables	Adjusted OR (95% CI) for END^*^	*p* Value	Adjusted OR (95% CI) for unfavorable outcome^*^	*p* Value
Per 1‐SD increase in LCN2	1.64 (1.20–2.25)	.002	1.73 (1.22–2.45)	.002
LCN2 quartiles				
First	Reference		Reference	
Second	4.35 (1.22–15.44)	.023	1.59 (0.64–3.93)	.317
Third	3.89 (1.09–13.89)	.037	2.19 (0.90–5.30)	.082
Fourth	5.16 (1.46–18.23)	.011	3.07 (1.25–7.56)	.015

Abbreviations: CI, confidence interval; END, early neurological deterioration; OR, odds ratio.

^*^Multivariate logistic regression analysis was adjusted for age, sex, reperfusion therapy, and variables with a *p* value <.1 in the univariate analysis.

**FIGURE 2:**
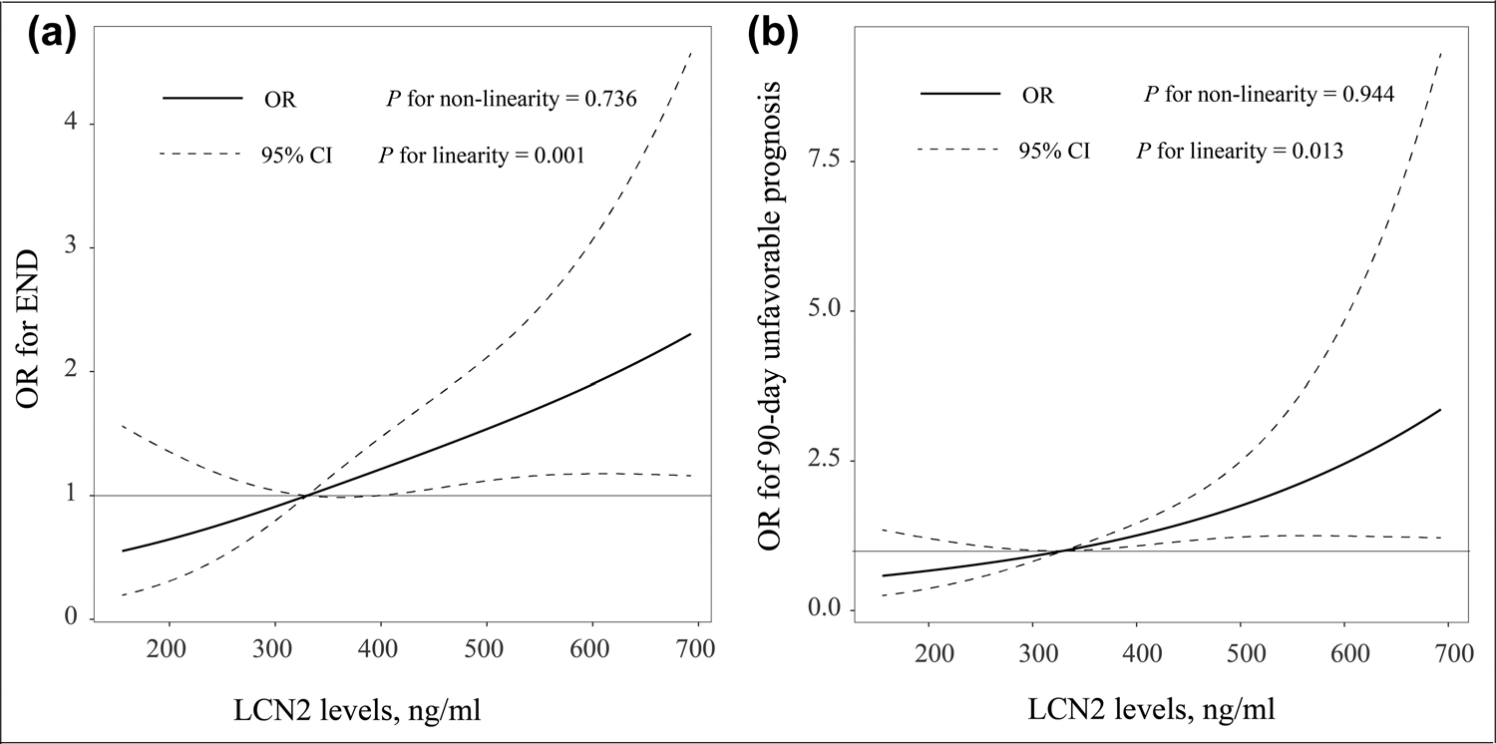
Association of serum lipocalin‐2 (LCN2) levels with risk of early neurological deterioration (END) (A) and 90‐day unfavorable outcome (B). Odds ratios and 95% confidence intervals were derived from restricted cubic spline regression, with knots placed at the 5th, 50th, and 95th percentiles of the distribution of LCN2. The reference point for serum LCN2 is the midpoint of the reference group from the categorical analysis. Odds ratios were adjusted for age, sex, and variables with a *p* value <.1 in the univariate analysis.

The pattern and magnitude of the relationship between LCN2 levels and clinical outcomes are shown in Figure 2. The multiple‐adjusted spline regression model displayed a linear association of LCN2 with risk of END (*p* = .001 for linearity) and 90‐day poor outcome (*p* = .013 for linearity).

## 4 DISCUSSION

In this cohort study of 253 subjects with ischemic stroke, we demonstrated that baseline circulating LCN2 levels were positively associated with the development of END and 90‐day poor functional outcome, which remained statistically significant after adjustment for important prognostic covariates of stroke.

Previous investigations on END used different definitions, leading to a discrepancy in the incidence rates. In this study, we defined the END as an NIHSS increment ≥4 points within 72 h after admission, which is the most commonly used definition (Siegler & Martin‐Schild, 2011; Sun et al., 2014; Thanvi et al., 2008). As a result, 16.2% of patients were diagnosed with END, which was similar to previous data (Siegler & Martin‐Schild, 2011; Thanvi et al., 2008; Zhang et al., 2016). In addition, the inhospital mortality rate was 7.2%, which is slightly higher than that previously reported in a study from the big data observatory platform for stroke of China (death/discharge against medical advice: 6.2%) (Tu et al., 2021). This discrepancy might due to the difference in study sample and methods.

During the past few years, LCN2 was considered an attractive blood‐based biomarker of inflammation and ischemia. Clinical studies have confirmed an increased level of LCN2 in the serum of patients with mild cognitive impairment (Choi et al., 2011) and in the cerebrospinal fluid of patients with multiple sclerosis (Al Nimer et al., 2016). Furthermore, elevated circulating LCN2 levels have been linked to an increased risk of cardiovascular disease (Cheng et al., 2014; Wu et al., 2014). Although the above studies are supportive of the role of LCN2 in the pathogenesis of central nervous system disease, few data are available detecting the LCN2 in association with secondary brain injury and stroke morbidity in ischemic stroke patients. In our study, we were able to support the assumption that LCN2 has the potential to predict early outcomes after ischemic stroke. The mechanisms underlying the association between LCN2 and neurological deterioration after stroke are incompletely clear. However, some hypothetical causes might lead to functional disability. First, LCN2 could regulate the blood–brain barrier integrity. LCN2 was reported to reduce MMP‐9 degradation and prolong its activity, thereby augmenting the deleterious effects of MMP‐9 on the blood–brain barrier (Turner & Sharp, 2016). LCN2 also induces the expression of vascular endothelial growth factors, which could affect vascular permeability either directly or via astrocytes (Kim et al., 2017). During the acute phase after stroke, immune cells could infiltrate into the ischemic hemisphere through the blood–brain barrier and promote brain tissue damage (Wang et al., 2020). Second, LCN2 was involved in the expression of pro‐inflammatory cytokines, chemokines, and adhesion molecules after ischemic stroke (Jin et al., 2014; Wang et al., 2015). LCN2 could activate its receptor of 24p3R and promote the cellular release of the high mobility group box 1, which could subsequently aggravate oxidative stress and NLRP3 inflammasome activation (Mondal et al., 2020). Therefore, these findings suggest that LCN2 may induce neurological deterioration by mediating oxidative stress and neuroinflammation after stroke. Finally, iron overload after stroke may induce perihematomal edema and brain injury (Keep et al., 2012). LCN2 may function as a mediator of iron homeostasis as it is capable of transporting iron into cells through the siderophore (Devireddy et al., 2005). However, there are some controversies regarding the regulation of intracellular iron concentration by LCN2. For example, in the sepsis model of LCN2‐deficient mice, intracellular labile iron was elevated (Srinivasan et al., 2012). The inconsistency between these data may be due to differences in methodologies. The role of LCN2 in iron transport after ischemic stroke requires further study.

Several caveats should be considered when explaining the present results. First, this was a single‐center study with a relatively small sample size, which limits the generalization to other groups of subjects. Second, the circulating LCN2 level was measured only once after admission, we therefore unable to evaluate the dynamic changes of LCN2 after stroke. Third, although several confounders in the multivariate analysis were controlled, there is also a possibility of residual confounding in our study. Finally, data were observational, which cannot establish causation. We, therefore, recommend a prospective validation in a larger cohort to verify our findings.

## 5 CONCLUSION

In summary, this study performed in the Chinese population demonstrated that increased serum LCN2 may be an independent predictor of END and 90‐day poor outcome after ischemic stroke. Further studies are warranted to determine the pathophysiological role of LCN2 in mediating stroke outcomes.

## AUTHOR CONTRIBUTIONS

Yi Xie, Xingfeng Zhuo, and Kai Xing designed the research and wrote the manuscript. Zhenqian Huang, Hongquan Guo, and Pengyu Gong carried out the data collection and follow‐up. Xingfeng Zhuo performed the data curation and review. Xingfeng Zhuo and Yun Li supervised the study. All authors have made an intellectual contribution to the manuscript and approved the submission.

## CONFLICT OF INTEREST STATEMENT

All the authors declare that there is no conflict of interest.

### PEER REVIEW

The peer review history for this article is available at https://publons.com/publon/10.1002/brb3.2979.

## Data Availability

The data that support the findings of this study are available on request from the corresponding author.
